# Safety Evaluation of Potential Toxic Metals Exposure from Street Foods Consumed in Mid-West Nigeria

**DOI:** 10.1155/2017/8458057

**Published:** 2017-04-26

**Authors:** O. C. Ekhator, N. A. Udowelle, S. Igbiri, R. N. Asomugha, Z. N. Igweze, O. E. Orisakwe

**Affiliations:** ^1^Department of Experimental Pharmacology & Toxicology, Faculty of Pharmacy, University of Port Harcourt, Rivers State, Nigeria; ^2^Department of Chemistry, Faculty of Science, Nnamdi Azikiwe University, Awka, Anambra State, Nigeria; ^3^Faculty of Pharmacy, Madonna University Elele, Port Harcourt, Rivers State, Nigeria

## Abstract

*Objective.* Street-vended foods offer numerous advantages to food security; nevertheless, the safety of street food should be considered. This study has investigated the level of potential toxic metal (Pb, Cd, Hg, Sb, Mn, and Al) contamination among street-vended foods in Benin City and Umunede.* Methods.* Twenty street food samples were purchased from vendors at bus stops. Metals were analyzed with atomic absorption spectrophotometry. The methods developed by the US EPA were employed to evaluate the potential health risk of toxic metals.* Results.* The concentrations of the toxic metals in mg/kg were in the range of Pb (0.014–1.37), Cd (0.00–0.00017), Hg (0.00–0.00014), Sb (0.00–0.021), Mn (0.00–0.012), and Al (0.00–0.22). All the toxic metals except Pb were below permissible limit set by WHO, EU, and USEPA. The daily intake, hazard quotient, and hazard index of all toxic metals except for Pb in some street foods were below the tolerable daily intake and threshold value of 1, indicating an insignificant health risk. Total cancer risk was within the priority risk level of 1.0*E* − 04 but higher than the acceptable risk level of 1*E* − 06.* Conclusion.* Consumption of some of these street foods is of public health concern.

## 1. Introduction

In Nigeria and all over the world, millions of people daily consume street foods that provide a wide range of essential energy needs and nutrients. According to a survey by the World health Organization (WHO) in 1996, 74% of countries reported that street foods contribute significantly to urban food supply [1], defined as “ready-to-eat foods,” processed or fresh, which are sold at stationary locations or hawked in streets and open places as opposed to stores and licensed establishments [2]. These foods are mostly cheap and they are also easily accessible [3–5]. There is a wide acceptability of street-vended foods among majority of the Nigerian population which cuts across social, cultural, and political class. The inexpensive and convenient nature of these hawked foods has experienced an economic boom in the last decade.

Although street food offers numerous advantages in improving food security, the safety of these foods should be properly monitored. Food safety is the assurance that food will not cause any harm or deleterious effect to the consumer when it is consumed [1]. Street foods can pose a possible health risk to habitual consumers because food prepared and exposed to the open air for sale may become contaminated by pathogenic microorganism as well as chemical toxicant [6]. Contamination of street food is as a result of so many factors such as preparation methods, poor packaging, vehicular exhaust emission, indiscriminate waste disposal, poor sanitation, industrial emission, and a list of all other pollution sources in the surrounding vending area [7].

This is possible as vending sites are usually within congested streets characterised by heavy vehicular traffic and industrial or commercial activities.

Recent researches have shown that potential toxic metals are now one of the major food and dietary contaminants in southern Nigeria [8, 9]. A number of serious health issues can develop as a result of excessive uptake of potential toxic metals such as lead, cadmium, mercury, aluminium, manganese, and antimony from dietary sources. Furthermore, excessive depletion of essential nutrients in the body can occur as a result of consumption of potential toxic metal-contaminated foods, causing a decrease in immunological defences, intrauterine growth retardation, impaired psychosocial behaviors, disabilities associated with malnutrition, and a high prevalence of upper gastrointestinal cancer [9, 10].

Continuous ingestion of heavy metals can have a damaging effect on humans and animals [11, 12]. Heavy metals like chromium, copper, manganese, and zinc reportedly can cause noncarcinogenic hazards such as neurologic disorders and liver disease when they are present in food in concentrations above their safe levels [13]. Consumption of rice and other foods contaminated with cadmium were associated with an increased risk of menopausal breast cancer [14]. Heavy metals induced toxicity is of public health concern, since food ingestion is the major pathway for human exposure to toxic substances. Therefore, there is the need to monitor their levels in frequently consumed street foods marketed in Nigerian cities. This study is aimed at investigating the levels of potential toxic metals (Pb, Cd, Hg, Sb, Mn, and Al) and also calculating the dietary daily intake as well as the hazard quotient and cancer risk in street foods commonly consumed in Benin City and Umunede, Nigeria, to assess their health risk.

## 2. Materials and Methods

### 2.1. Collection of Samples

**Figure 1:**
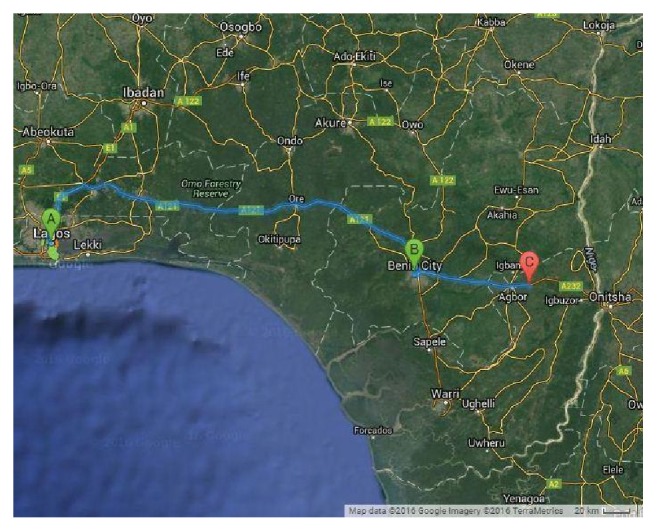
Google map of Nigeria showing Lagos (A), Benin City (B), and Umunede (C), Onitsha highway.

In February 2016, twenty different varieties of commonly consumed street food samples such as jollof rice, spaghetti, beans, roasted plantain, fried chicken, fried turkey, fried meat, fried fish, doughnut, meat pie, edible maggot, roasted yam, buns, fried plantain, white rice, stewed meat, salad, cake, sausage, and moi-moi were purchased from major bus stops at different locations in Benin City and Umunede, Mid-Western Nigeria (Figure 1), directly from the vendors. The food samples were collected in glass Petri dish. Three samples of a particular variety were collected from the different locations. The foods were cut into small pieces and kept in tightly sealed glass Petri dish in the refrigerator for subsequent ashing, digestion, and analysis.

### 2.2. Determination of Potential Toxic Metals

#### 2.2.1. Digestion of Samples

Five grams of each food sample was digested in 9 mL of 65% concentrated HNO_3_ and 3 mL perchloric acid. The solution was transferred to a hot plate of 110°C for about 5 hours. Afterwards, the samples were introduced into an oven under a temperature that was gradually increased in 100°C every 60 minutes until the wished final temperature of 450°C was reached 18 hours later; white ashes were obtained. Following this, samples were left to cool. The white ashes were then dissolved with 1.5% HNO_3_ (5 mL) and a final volume of 25 mL was made by adding deionized water. The resulting solution was filtered using a Whatman filter paper (number 42) fitted into a Bucher funnel into a beaker before it was transferred into a tightly sealed plastic container.

### 2.3. Analysis

The presence of Pb, Cd, Hg, Sb, Mn, and Al was analyzed in samples using a Solaar Thermo Elemental Flame Absorption Spectrometer (S4 710).

### 2.4. Quality Control

The instrument was recalibrated after every ten runs. The analytical procedure was checked using spike recovery method (SRM). A known standard of the metals was introduced into already analyzed samples and reanalyzed. The results of the recovery studies for Pb, Cd, Hg, Sb, Mn, and Al were more than 95%. The relative standard deviation between replicate analyses was less than 4%. The limit of detection (LOD) for Cd, Hg, Sb, Mn, and Al was 0.005 whereas the LOD of Pb was 0.01 ppm, with blank values reading as 0.00 ppm for all the metals in deionized water with electrical conductivity value of lower than 5 *μ*S/cm. The limits of quantification LOQ for Cd and Sb were 0.004 and for Hg, Mn, and Al were 0.006 ppm. Two-way analysis of variance (ANOVA) and a Student's *t*-test were used to determine whether the concentrations of the metals varied significantly, with values less than 0.05 (*p* < 0.05) considered to be statistically significant. The statistical calculations were performed with Graph Pad Prism 5.0.

## 3. Risk Assessment Methods

The human health risk models including carcinogenic and noncarcinogenic developed by US EPA have proved successful and been adopted worldwide. Currently, there is no agreed limit for acceptable maximum carcinogenic and noncarcinogenic risk levels in Nigeria. We therefore employed the US EPA model and its threshold values to assess the potential human health risks posed by heavy metal contamination in this study.

### 3.1. Estimated Daily Intake

The daily intake of metals depends on the metal concentration in food, the daily food consumption, and the body weight. The estimated daily intake (EDI) of metals is a concept introduced to take into account these factors. The EDI was calculated based on the following formula [15]:

(1) $$\begin{array}{c} EDI=\frac{C_{metal}\times D_{food\;intake}}{{BW}_{average}}, \end{array}$$

where *C* is the metal concentration in the street food in mg/kg, *D* is the daily intake of food in kg person^−1^, and BW is average body weight in kg (70 kg adults, 24 kg children).

### 3.2. Noncarcinogenic Risk

#### 3.2.1. Target Hazard Quotient (THQ)

Noncarcinogenic risk estimation of heavy metals consumption was determined using THQ values. THQ is a ratio of the determined dose of a pollutant to a reference level considered harmful. THQ values were determined based on the following formula [15]:

(2) $$\begin{array}{c} THQ=\frac{Efr\times ED\times FIR\times C}{RfDo\times B_{average\;wt}\times ATn\times 10^{-3}}, \end{array}$$

where Efr is exposure frequency assumed to be 365 days year^−1^, ED is exposure duration in 56 years equivalent to an average lifetime, FIR is average daily consumption in kg person^−1^day^−1^, *C* is concentration of metal in food sample in mg/kg, RfDo is reference dose in mg/kg day^−1^, and ATn is average exposure time for noncarcinogens in days.

#### 3.2.2. Chronic Hazard Index (HI)

The chronic hazard index (HI) is the sum of more than one hazard quotient for multiple toxicants or multiple exposure pathways; it was calculated using the equation below:

(3) $$\begin{array}{c} HI=\sum THQ. \end{array}$$

### 3.3. Carcinogenic Risk

Cancer risk can be evaluated from

(4) $$\begin{array}{c} Cancer\;\;risk=CDI\times SF, \end{array}$$

where cancer risk represents the probability of an individual lifetime health risks from carcinogens; CDI is the chronic daily intake of carcinogens (mg kg^−1^ d^−1^); SF is the slope factor of hazardous substances (mg kg^−1^ d^−1^).

The cumulative cancer risk can be calculated from

(5) $$\begin{array}{c} Total\;\;cancer\;\;risk=\overset{n}{\underset{k=1}{\sum }}CDI_{k}SF_{k}, \end{array}$$

where CDI_*k*_ is the chronic daily intake (mg kg^−1^ d^−1^); of substance *k*, SF_*k*_ is the slope factor for substance *k* (mg kg^−1^ d^−1^). The acceptable or tolerable risk for regulatory purposes is within the range of 10^−6^–10^−4^ [16].

## 4. Results

Table 1 shows the level of potential toxic metals (Pb, Cd, Hg, Sb, Mn, and Al) in street foods: jollof rice, spaghetti, beans, roasted plantain, fried chicken, fried turkey, fried meat, fried fish, doughnut, meat pie, edible maggot, roasted yam, buns, fried plantain, white rice, stewed meat, salad, cake, sausage, and moi-moi. The concentration (mg/kg) of the various metals in the street food samples ranged within 0.00–1.37 mg/kg in Pb (0.014–1.37), Cd (0.00–0.0017), Hg (0.00–0.0014), Sb (0.00–0.021), Mn (0.00–0.012), and AI (0.00–0.225) all in mg/kg, respectively. The highest levels of heavy metals concentration were detected in Pb (beans, 1.37 mg/kg), Cd (stewed meat, 0.0017), Hg (stewed meat, 0.0014), Sb (fried fish, 0.021), Mn (fried meat, 0.012), and AI (stewed meat, 0.221 mg/kg), respectively. Pb was detected in 100% of the investigated street food samples, 70% of the street food samples showed Cd levels which were below the limit of detection (<0.001), and also 80% of the studied samples had Hg levels which were below the detectable limit. Mn and Al were observed to be below the detectable limit in 65% and 10% of street food samples, respectively.

Table 2 shows the estimated daily intake (EDI) of heavy metals for an individual with a body weight of 70 kg and 24 kg for adults and children, respectively. The daily intake of Pb from street food consumption for both adults and children ranged within 0.00004–0.0069 and 0.000072–0.011 mg/kg bw day^−1^, respectively. The highest intake of Pb was from beans consumption with a daily intake of 0.0069 and 0.011 mg/kg bw day^−1^ for adults and children, respectively. The estimated daily intake of Cd from street food for adults and children ranged within 1.30*E* − 6–4.50*E* − 6 and 2.2*E* − 6–9.9*E* − 6 mg/kg bw day^−1^, respectively. The stewed meat had the highest daily intake of cadmium with a daily intake rate of 4.50*E* − 6 and 9.90*E* − 6 for adults and children, respectively. Daily intake rate of Hg ranged within 2.60*E* − 7–3.70*E* − 6 and 5.80*E* − 7–8.17*E* − 6 for adults and children. Stewed meat with an intake rate of 3.7*E* − 6 and 8.17*E* − 6 for both adults and children contributed the highest daily intake of Hg. Also the consumption of fried meat and white rice with an EDI of 5.70*E* − 6 was observed to be the source for highest intake of Sb in adults while the highest intake of Sb for children was seen in fried meat (1.20*E* − 4). The EDI of Mn from street food samples ranged within 9.2*E* − 6–5.7*E* − 4 mg/kg bw/day in adults and 1.3*E* − 5–1.3*E* − 3 mg/kg bw/day in children; the highest value was found in stewed meat for both adults and children with intake values of 5.7*E* − 4 and 1.3*E* − 3 mg/kg bw/day, respectively. Table 2 shows the estimated daily intake (EDI) of heavy metals for an individual with a body weight of 70 kg and 24 kg for adult and children, respectively. The daily intake of Pb from street food consumption for both adults and children ranged within 0.00004–0.0069 and 0.000072–0.011 mg/kg bw day^−1^, respectively. The highest intake of Pb was from beans consumption with a daily intake of 0.0069 and 0.011 mg/kg bw day^1^ for adults and children, respectively. The estimated daily intake of Cd from street food for adult and children ranged within 1.30*E* − 6–4.50*E* − 6 and 2.2*E* − 6–9.9*E* − 6 mg/kg bw day^−1^, respectively. The stewed meat had the highest daily intake of cadmium with a daily intake rate of 4.50*E* − 6 and 9.90*E* − 6 for adults and children, respectively. Daily intake rate of Hg ranged within 2.60*E* − 7–3.70*E* − 6 and 5.80*E* − 7–8.17*E* − 6 for adults and children. Stewed meat with an intake rate of 3.7*E* − 6 and 8.17*E* − 6 for both adults and children contributed the highest daily intake of Hg. Also the consumption of fried meat and white rice with an EDI of 5.70*E* − 6 was observed to be the source for highest intake of Sb in adults while the highest intake of Sb for children was seen in fried meat (1.20*E* − 4). The EDI of Mn from street food samples ranged within 9.2*E* − 6–5.7*E* − 4 mg/kg bw/day in adults and 1.3*E* − 5–1.3*E* − 3 mg/kg bw/day in children; the highest value was found in stewed meat for both adults and children with intake values of 5.7*E* − 4 and 1.3*E* − 3 mg/kg bw/day, respectively.

The target hazard quotient (THQ) of each metal through consumption of street foods sold in the vicinity of Benin for both adults and children increased in the following order: Al < Mn < Hg < Cd < Sb < Pb, as shown in Table 3. The maximum value of THQ was seen in jollof rice (0.39 and 0.65) for adults and children in Pb. The THQ values of other street foods varied from Pb (0.0092–0.3896), Cd (0.0026–0.0034), Hg (0.0009–0.0026), Sb (0.0081–0.1414), Mn (0.0002–0.0023), and Al (6.6*E* − 5–1.9*E* − 3), respectively, for adults and also for children ranged from Pb (0.0181–0.6526), Cd (0.0057–0.0076), Hg (0.0019–0.0058), Mn (0.0110–0.3121), Sb (0.0008–0.0029), and Al (0.0001–0.0039), respectively. The THQ values for street food consumption from Benin City were less than 1. The THQ of heavy metals from street food in Umunede increased in the following order: Mn < Al < Cd < Hg < Sb and Pb, with values ranging between Pb (0.0689–1.7143), Cd (0.0031–0.0045), Hg (0.0053–0.0123), Sb (0.0093–0.1425), Mn (0.0003–0.0008), and Al (0.0001–0.0041), respectively, for adults and ranging from Pb (0.0848–2.8714), Cd (0.0002–0.0099), Hg (0.0087–0.02720), Sb (0.0087–0.2387), Mn (0.0005–0.0018), and Al (0.0002–0.0025), respectively, for children. THQ values for Pb in street foods beans (1.7143), white rice (1.2908), and moi-moi (1.5459) for adults and Pb THQ values for beans (2.8714), white rice (2.162), moi-moi (2.5893), and stewed meat (1.9581) for children were higher than 1.

Table 3 also shows the hazard index of mixtures of metal intake from street food for adults and children. In children the HI value was highest in beans (2.9) and least in roasted yam (0.09) while for adults HI value was highest in beans (1.73) and least in buns (0.09) as seen in Table 3.

## 5. Discussion

Heavy metals are considered as one of the most important constituents of food contamination from the environment due to its ability to persist, accumulate, and become toxic to living organism through consumption [17]. This present study is a risk assessment study of lead (Pb), cadmium (Cd), mercury (Hg), antimony (Sb), manganese (Mn), and aluminium (Al) in different street-vended foods sold in Benin City and Umunede of Mid-Western Nigeria; the trend of heavy metals contamination in the street food sample decreased in the following order: Pb > Al > Sb > Mn > Cd > Hg.

Lead was detected in all the street food samples, with 100% of street food samples seen to be higher than 0.01 mg/kg which is the maximum permissible limit set by WHO/FAO and also the maximum allowable concentration of 0.02 mg/kg by EU and 0.05 mg/kg limit set by USEPA; Pb levels were also in violation of these standards except for fried chicken and edible maggot which were below 0.05 mg/kg and therefore within the acceptable limit. The high percentage of food samples which were in violation of the maximum permissible limits of Pb set by WHO, EU, and US EPA is a cause for public health concern considering the frequency of exposure. The consumption of food contaminated with Pb is the major source of exposure to Pb in a general population [18].

These values were higher than the Pb values reported by Bordajandi et al. [19] in food samples from Huelva (Spain). High concentration burden of Pb in the body can cause irreversible brain damage (encephalopathy), anemia, coma, and death if not treated immediately [20]. Long-term system exposure can cause damage to the kidneys and reproductive and immune system. Children are more vulnerable than adults to the toxic effects of Pb and they also absorb Pb easily. A low blood lead level can affect the intellectual development or IQ of young children [21]. Cadmium is classified by the IARC as a group 1 cancer causing agent. It has been shown that chronic exposure to low doses of cancer causing heavy metals may be implicated in various types of cancer. In the current study, the levels of cadmium detected in street food samples were all below the permissible limit of Cd set by US EPA, WHO, and EU (0.05 mg/kg). Although most samples contaminated with Cd were below the detectable limit, Cd was detected in 30% of the street food samples which were mainly meat based food products such as fried meat, fried chicken, fried turkey, edible maggot, and stewed meat. With a half-life of 10 years, this trend of Cd contamination on mostly meat samples could be as a result of bioaccumulation of Cd in the animals. Dietary Cd intake due to the consumption of environmentally contaminated rice and other foods was associated with an increased risk of postmenopausal breast cancer [14]. Mercury showed the least level of contamination in street foods, detected in 20% of the samples ranging between 0.0003 and 0.0014 in predominantly meat based food products which include stewed meat, fried meat, fried fish, and fried turkey. The concentration of Hg in street food samples was below the food safety limits. Inorganic mercury in aquatic environment is transformed by microorganism into methyl mercury which is lipophilic and biomagnify in the food chain. Mothers who are exposed to Hg through their diet pass the toxicant to their fetus and to infants through breast milk [22]. This makes the fetus and children more vulnerable to low level mercury leading to toxicity. Decreased performance in area of motor functions and memory loss has been reported among children exposed to presumably safe mercury level. Similarly, disruption of attention, fine motor function, and verbal memory was also found in adult on exposure to low mercury level [23]. Mercury has been found to be a causative agent of various sorts of disorders, including neurological [24], renal [25], reproduction [26], genetic [27], cardiac [28], and immunological [29] disorders.

Manganese is an essential trace metal for animal with essential enzymes activities in the biochemical processes of the body. However, in large quantities the metal can cause acute and chronic poisoning. Manganese was detected in 35% of street food samples with concentrations ranging between 0.0011 and 0.01 mg/kg which is lower than the guideline value of 0.16 mg/kg [30]. Mn can be neurotoxic when exceeding the homeostatic range [31]. Mn exposure is associated with cognitive, motor, and behavior deficits in children.

Aluminium (Al) and antimony (Sb) are also among the potential toxic metals that have no biological function in the body. Al and Sb are considerably less toxic than either Hg or Pb but can be toxic at higher levels. In this study Al and Sb were detected in 90% and 75% of the street food samples, respectively; their ranges were Al (0.00–0.23 mg/kg) and Sb (0.00–0.021) mg/kg. Al and Sb values were below the permissible limit of 1 mg/kg for both metals (ATSDR 1999). Higher level of Al has the potential to cause a number of health problems such as neuromuscular disorder, osteomalacia, Parkinson's disease, autism, Alzheimer's disease, and autoimmunity [32]. Antimony toxicity is characterised by gastrointestinal symptoms, vomiting, kidney damage, and so forth [33].

To assess the risk of heavy metal exposure to human health in the exposed population, information about the dietary intake is necessary. Tolerable daily intake (TDI) is an estimate of daily exposure to the human population that is likely to be without an appreciable risk of adverse effect during a life time. In this study the estimated daily intake of Pb for adult and children was in the range of 0.00004–0.0069 mg/kg day and 0.000072–0.011 mg/kg/day, respectively. Some of the street foods were in violation of the tolerable daily intake for a 70 kg individual and children with a lower body weight which had higher intake of Pb and this is of health risk concern. Amongst the street foods that violated the tolerable daily intake (TDI) of Pb for adults and children were stewed meat, white rice, beans, and moi-moi. This observation is noteworthy and of significant public health importance in both children and adult since the food items are usually taken together as a meal. Daily intake of these street foods may likely cause significant health hazard to the residents. For the other potential toxic metals such as Cd, Hg, Sb, Mn, and Al their estimated daily intake was below the tolerable daily intake for both adults and children set by FAO, WHO, and US EPA. The calculated THQ values of potential toxic metals for both adult and children in the present study presented a public health concern with THQ values > 1 in some samples. The THQ of Pb in white rice, beans, and moi-moi were above 1 for adults, while for children stewed meat, white rice, beans, and moi-moi also had values greater than 1 and higher than the values in adults. Children are more susceptible to heavy metal poisoning than adults due to their reduced body weight, hence the reason for increased toxicity. These THQ values are an indication that street food samples which had values > 1 are unsafe for human consumption. The THQ for Cd, Hg, Sb, Mn, and Al can be considered to be safe for the consumers due to their low noncarcinogenic risk presenting values < 1.

Hazard index which was calculated to represent the combined risk of heavy metal toxicity is the sum total of all the THQ values in a food sample and a value > 1 is an indication that the probability of an adverse health effect associated with such exposure is high. For adults the HI value due to the consumption of white rice, beans, and moi-moi was greater than 1 while for children stewed meat, white rice, beans, and moi-moi had a higher HI value when compared to adults. Dietary combinations (jollof rice in combination with salad and fried meat or white rice combined with fried plantain and fried chicken) which are common in Nigeria may increase the exposure risk and possibility of metal toxicity.

Carcinogenic risk is estimated and expressed as a probability of contracting cancer over a lifetime of 70 years. The cancer risk decreased in the order of Pb > Cd. The total cancer risk for adults and children, respectively, was 5.1*E* − 05 and 8.6*E* − 05 (Benin City) and 2.2*E* − 04 and 3.7*E* − 04 (Umunede); these ranges were seen to be within the priority risk level of 1.0*E* − 04 but higher than the acceptable risk level of 1*E* − 06 [34]. This suggests that, for toxic metals Pb and Cd, the carcinogenic risk through consumption of street foods for both adult and children is of public health concern. The sources of potential toxic metals contamination in these street foods may be at the points of handling and processing/cooking given the heavy vehicular traffic and sundry activities characteristic of these vending areas. The source of the raw food (farm produce and poultry) may be of concern too. Mindful of only making profit, vendors may purchase cheap and substandard, raw foods from unsafe environments (e.g., fish caught in waters where fishing is prohibited and vegetables or rice that are cultivated in heavily polluted soils) [35]. Street foods may also be contaminated by chemical toxicant due to leaching from cooking utensils, roasting gauzes, and packaging materials. Location of street food vendors in congested streets and bus or train stations exposes the food to airborne pollutant deposition from automobile exhaust fumes [36].

## 6. Conclusion

Taken together all the potential toxic metals concentrations and daily intake except Pb in this study were lower than the limit established by the EU, WHO, and USEPA. The daily intake, THQ, and HI of Pb in some street food samples were higher than the established limit, an indication of health risk among the exposed population. Foodstuffs contamination by heavy metals is unavoidable as a result of their presence in the environment (air, water, and soil) and this can be detrimental to human health due to their ability to accumulate in body tissues.

## Figures and Tables

**Table 1:** Concentration (mg/kg) of heavy metals in commonly consumed street foods marketed in Benin City and Umunede, Mid-Western Nigeria.

Location/samples	Pb	Cd	Hg	Sb	Mn	Al
*Benin City *						
Salad	0.14	<0.001	<0.001	<0.001	<0.001	0.003
Jollof rice	0.31	<0.001	<0.001	0.0013	<0.001	0.007
Meat pie	0.24	<0.001	<0.001	0.013	0.01	0.011
Doughnut	0.21	<0.001	<0.001	0.002	<0.001	0.003
Cake	0.12	<0.001	<0.001	<0.001	<0.001	<0.001
Sausage	0.14	<0.001	<0.001	0.0012	<0.001	<0.001
Fried meat	0.31	0.0012	0.0001	0.02	0.012	0.034
Fried chicken	0.026	0.0013	<0.001	0.012	0.0019	0.012
Fried turkey	0.14	0.001	0.0003	0.0114	0.0011	0.024
Edible maggot	0.014	0.0011	<0.001	0.0029	0.0033	0.104
*Umunede *						
Fried fish	0.2	0.001	0.0012	0.021	0.0032	0.135
Stewed meat	1.34	0.0017	0.0014	0.0021	0.0044	0.22
Roasted plantain	0.31	<0.001	<0.001	0.0021	<0.001	0.017
Roasted yam	0.13	<0.001	<0.001	0.0013	<0.001	0.043
Spaghetti	0.24	<0.001	<0.001	<0.001	<0.001	0.013
White rice	1.033	<0.001	<0.001	0.0114	<0.001	0.004
Beans	1.37	<0.001	<0.001	0.0014	<0.001	0.042
Buns	0.14	<0.001	<0.001	<0.001	<0.001	0.022
Moi-moi	1.24	<0.001	<0.001	<0.001	<0.001	0.0103
Fried plantain	0.24	<0.001	<0.001	0.0023	<0.001	0.0114

<0.001: below detectable limit.

**Table 2:** Estimated daily intake of heavy metals (mg/kg bw/day) for adults (70 kg) and children (24 kg) from consumption of street-vended foods sold in Benin City and Umunede.

Location	Adult 70 Kg						Children 24 kg					
	Pb	Cd	Hg	Sb	Mn	Al	Pb	Cd	Hg	Sb	Mn	Al
*Benin City *												
Salad	0.0005	ND	ND	ND	ND	1.0*E *− 5	7.3*E *− 4	ND	ND	ND	ND	1.6*E *− 5
Jollof rice	0.0016	ND	ND	6.5*E *− 6	ND	3.6*E *− 5	2.6*E *− 3	ND	ND	1.1*E *− 5	ND	5.9*E *− 5
Meat pie	0.0007	ND	ND	3.5*E *− 5	2.8*E *− 5	3.1*E *− 5	9.6*E *− 4	ND	ND	5.1*E *− 5	4.1*E *− 5	4.5*E *− 5
Doughnut	0.0006	ND	ND	6.2*E *− 6	ND	9.2*E *− 6	8.3*E *− 4	ND	ND	9.1*E *− 6	ND	1.3*E *− 5
Cake	0.0003	ND	ND	ND	ND	ND	4.8*E *− 4	ND	ND	ND	ND	ND
Sausage	0.0004	ND	ND	3.3*E *− 6	ND	ND	5.6*E *− 4	ND	ND	4.8*E *− 6	ND	ND
Fried meat	0.0008	3.2*E *− 6	2.6*E *− 7	5.7*E *− 5	3.2*E *− 5	9.0*E *− 5	1.8*E *− 3	7*E *− 6	5.8*E *− 7	1.2*E *− 4	7*E *− 5	2*E *− 4
Fried chicken	7.0*E *− 5	3.4*E *− 6	ND	3.2*E *− 5	5.0*E *− 6	3.2*E *− 5	1.5*E *− 4	7.6*E *− 6	ND	7.5*E *− 5	1*E *− 5	7.1*E *− 5
Fried turkey	0.0004	2.6*E *− 6	7.9*E *− 7	3.0*E *− 5	2.9*E *− 6	6.4*E *− 5	8.2*E *− 4	5.8*E *− 6	1.8*E *− 6	6.7*E *− 5	6.4*E *− 6	1.4*E *− 4
Edible maggot	4.0*E *− 5	2.9*E *− 6	ND	7.7*E *− 6	8.7*E *− 6	2.7*E *− 4	7.2*E *− 5	5.7*E *− 6	ND	1.5*E *− 5	1.7*E *− 5	5.4*E *− 4
*Umunede*												
Fried fish	0.0003	1.3*E *− 6	1.6*E *− 6	2.8*E *− 5	4.2*E *− 6	1.8*E *− 4	4.5*E *− 4	2.2*E *− 6	2.2*E *− 6	4.6*E *− 5	6.9*E *− 5	2.9*E *− 4
Stewed meat	0.0035	4.5*E *− 6	3.7*E *− 6	5.6*E *− 6	1.2*E *− 5	5.7*E *− 4	7.8*E *− 3	9.9*E *− 6	8.2*E *− 6	1.2*E *− 5	2.6*E *− 5	1.3*E *− 3
Roasted plantain	0.0009	ND	ND	6.0*E *− 6	ND	4.8*E *− 5	8.3*E *− 4	ND	ND	5.6*E *− 6	ND	4.5*E *− 5
Roasted yam	0.0004	ND	ND	3.7*E *− 6	ND	1.2*E *− 4	3.4*E *− 4	ND	ND	3.5*E *− 6	ND	1.1*E *− 4
Spaghetti	0.0012	ND	ND	ND	ND	6.5*E *− 5	2.3*E *− 3	ND	ND	ND	ND	1.1*E *− 4
White rice	0.0052	ND	ND	5.7*E *− 5	ND	1.9*E *− 5	8.6*E *− 3	ND	ND	9.5*E *− 5	ND	3.1*E *− 5
Beans	0.0069	ND	ND	7.0*E *− 6	ND	2.1*E *− 4	1.1*E *− 2	ND	ND	1.2*E *− 5	ND	3.5*E *− 4
Buns	0.0004	ND	ND	ND	ND	5.9*E *− 5	5.7*E *− 4	ND	ND	ND	ND	8.6*E *− 5
Moi-moi	0.0062	ND	ND	ND	ND	5.2*E *− 5	1.0*E *− 2	ND	ND	ND	ND	8.6*E *− 5
Fried plantain	0.0007	ND	ND	6.6*E *− 6	ND	3.3*E *− 5	6.3*E *− 4	ND	ND	6.6*E *− 6	ND	3.0*E *− 5

**Table 3:** Target hazard quotient (THQ) and hazard index (HI) for adults and children exposed to street-vended foods contaminated with heavy metals in Benin City and Umunede, Mid-Western Nigeria.

Samples	Adult 70 Kg							Children 24 Kg						
	Pb	Cd	Hg	Sb	Mn	Al	HI	Pb	Cd	Hg	Sb	Mn	Al	HI
*Benin City*														
Salad	0.1139	ND	ND	ND	ND	7.1*E* − 5	0.11	0.1837	ND	ND	ND	ND	0.0001	0.18
Jollof rice	0.3896	ND	ND	0.0163	ND	2.5*E* − 4	0.41	0.6526	ND	ND	0.0272	ND	0.0004	0.68
Meat pie	0.1653	ND	ND	0.0882	0.002	2.2*E* − 4	0.26	0.2411	ND	ND	0.1286	0.0029	0.0003	0.37
Doughnut	0.1426	ND	ND	0.0156	ND	6.6*E* − 5	0.16	0.2079	ND	ND	0.0228	ND	1*E* − 4	0.23
Cake	0.0823	ND	ND	ND	ND	ND	0.08	0.12	ND	ND	ND	ND	ND	0.12
Sausage	0.0956	ND	ND	0.0081	ND	ND	0.1	0.1395	ND	ND	0.0119	ND	ND	0.15
Fried meat	0.2073	0.0032	0.0009	0.1414	0.0023	6.4*E* − 4	0.36	0.4575	0.007	0.0019	0.3121	0.005	0.0014	0.79
Fried chicken	0.0174	0.0034	ND	0.0799	0.0004	2.3*E* − 4	0.1	0.0385	0.0076	ND	0.1765	0.0008	0.0005	0.22
Fried turkey	0.0928	0.0026	0.0026	0.0753	0.0002	4.6*E* − 4	0.17	0.2049	0.0058	0.0058	0.1663	0.0005	0.001	0.38
Edible maggot	0.0092	0.0029	ND	0.0192	0.0006	1.9*E* − 3	0.03	0.0181	0.0057	ND	0.0378	0.0012	0.0039	0.07
*Umunede*														
Fried fish	0.0689	0.0031	0.0053	0.07	0.0003	1.3*E* − 3	0.15	0.1135	0.0022	0.0087	0.1154	0.0005	0.0021	0.24
Stewed meat	0.8871	0.0045	0.0123	0.0139	0.0008	4.1*E* − 3	0.92	1.9581	0.0099	0.0272	0.0306	0.0018	0.009	2.04
Roasted plantain	0.2233	ND	ND	0.015	ND	3.0*E* − 4	0.24	0.2079	ND	ND	0.014	ND	0.0003	0.22
Roasted yam	0.0909	ND	ND	0.0093	ND	9.0*E* − 4	0.1	0.0848	ND	ND	0.0087	ND	0.0008	0.09
Spaghetti	0.3033	ND	ND	ND	ND	5.0*E* − 4	0.3	0.5079	ND	ND	ND	ND	0.0008	0.51
White rice	1.2908	ND	ND	0.1425	ND	1.0*E* − 4	1.43	2.162	ND	ND	0.2387	ND	0.0002	2.4
Beans	1.7143	ND	ND	0.0175	ND	1.5*E* − 3	1.73	2.8714	ND	ND	0.0293	ND	0.0025	2.9
Buns	0.0976	ND	ND	ND	ND	4.0*E* − 4	0.09	0.1423	ND	ND	ND	ND	0.0006	0.14
Moi-moi	1.5459	ND	ND	ND	ND	4.0*E* − 4	1.55	2.5893	ND	ND	ND	ND	0.0006	2.59
Fried plantain	0.1694	ND	ND	0.0164	ND	2.0*E* − 4	0.19	0.1581	ND	ND	0.0153	ND	0.0002	0.17

ND = not detected.
